# Extensive micropustular *Tinea capitis* in an adult caused by *Trichophyton verrucosum* with evolution to Kerion Celsi^⋆^

**DOI:** 10.1016/j.abd.2023.04.005

**Published:** 2023-11-18

**Authors:** Hiram Larangeira de Almeida Jr., Luiz Roberto Kramer Costa, Augusto Scott da Rocha

**Affiliations:** a Department of Dermatology, Universidade Federal de Pelotas, Pelotas, RS, Brazil; b Department of Mycology, Laboratório Ary Costa, Pelotas, RS, Brazil; c Faculty of Medicine, Universidade Federal de Pelotas, Pelotas, RS, Brazil

Dear Editor,

*Tinea capitis* is a fungal infection that affects mainly school-age children and can present different degrees of inflammation, with the inflammatory type known as *Kerion Celsi* being the most severe of all. Among the etiological agents causing this infection, *Trichophyton verrucosum* is an ectothrix zoophilic dermatophyte, commonly found in cattle, especially in young cattle, but rarely associated with cases of *tinea capitis* in human beings - especially in adults.^1^

*Trichophyton verrucosum* infection is almost invariably transmitted through contact with infected cattle. Despite the large herd of cattle in the country and reports of infection in these animals caused by this dermatophyte, there have been few reports in Brazil of *tinea capitis* in humans caused by *Trichophyton verrucosum*. The majority of the reported cases in humans come from Europe and Asia.

A 64-year-old immunocompetent patient, working in the rural environment, presented follicular pustules in the temporo-occipital region, which extended through almost the entire scalp (Fig. 1A). No hyphae were found in two microbiological examinations, only cocci, with negative bacterial and fungal cultures. He was on antibiotics (clindamycin and ceftriaxone) with no response. After ten days, lesions suggestive of *kerion celsi* appeared in the initial area (Fig. 1B). A third direct mycological examination demonstrated hyphae (Fig. 2A) and the culture identified *Trichophyton verrucosum* (Fig. 2B), with ocher-colored colonies, which grew at 37 °C and microscopically showed the characteristic rounded chlamydoconidia in strings (Fig. 2C). Scanning electron microscopy of a colony was performed and chain chlamydoconidia were also identified through this technique (Fig. 3).

**Figure 1:**
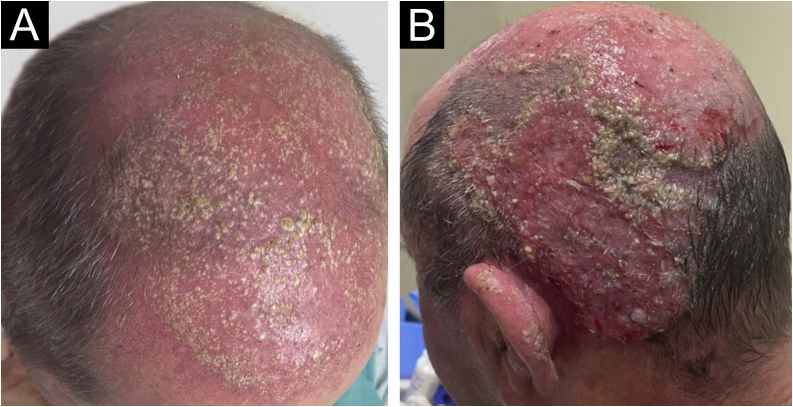
(A) Clinical aspect with follicular pustules. (B) Slightly vegetative area with pustules and crusts.

**Figure 2:**
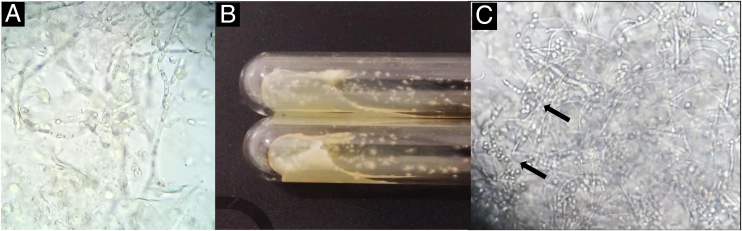
(A) Direct mycological examination with hyphae. (B) Culture examination with ocher-colored colonies. (C) Microscopic aspect of the culture with hyphae and rounded chlamydoconidia in strings (arrows).

**Figure 3:**
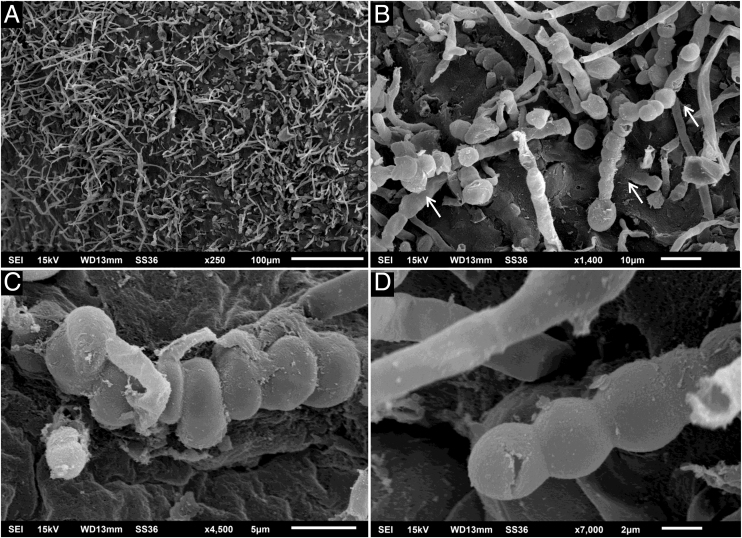
Scanning electron microscopy – (A) Low magnification showing filaments (×250). (B) Medium magnification showing rounded chlamydoconidia (arrows; ×1,400). (C‒D) High magnification detail of chlamydoconidia (×4.500 and ×7.000).

Therapy with terbinafine 250 mg a day was implemented for five weeks, followed by resolution of the condition, leaving residual erythema and alopecia (Fig. 4).

**Figure 4:**
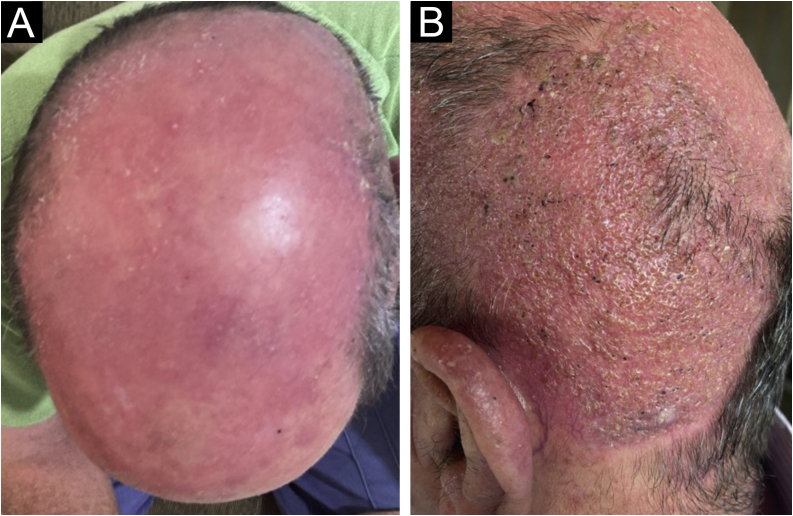
Clinical aspect after treatment: (A) with residual erythema; (B) with residual alopecia.

In Brazil, there have been rare reports of this agent, more frequently in the northeastern region, with 7.55% of cases in a publication of 82 patients with *tinea capitis*. In this series, 80% of the cases occurred before the age of 20.^2^ In a publication from Manaus, Amazonas, with 115 cases of *tinea capitis*, *T. verrucosum* was not identified in any of them.^3^ In a case series from Botucatu, São Paulo, with 364 positive culture tests for *tinea capitis*, *T. verrucosum* was not identified, either.^4^ From the central region of Rio Grande do Sul, there is a report of an average prevalence of this agent in around 1.4% of cases of *tinea capitis*.^5^ The reference laboratory for mycology in the southern region of Rio Grande do Sul has identified only three cases in 60 years, including the one reported herein, demonstrating the rarity of this etiological agent.^6^

In rural Ethiopia, the percentage of cases caused by *T. verrucosum* is much higher, reaching almost 30%,^7^ in contrast to information from other parts of the world, where there have been sporadic reports.^8^, ^9^

A study of 313 bovines, also from the region of the case reported herein, identified *T. verrucosum* in 95.8% of cattle with lesions suggestive of dermatophytosis, demonstrating its veterinary relevance. This study identified the agent in two animals (1,2%) with normal skin. It is noteworthy that it predominates in animals under six months of age, and just like human *tinea capitis*, it is rare in adult cattle. This high prevalence in animals suggests low infectivity of the agent for humans. Spontaneous cure has been reported, indicating the difficulty of *T. verrucosum* to develop in the non-ideal host.^6^

There are reports of difficulty in identifying the agent,^8^ as happened in this case, and it should be noted that it grows slowly and at a higher temperature than other dermatophytes and must be cultured in an incubator and not at room temperature.^1^, ^6^

This condition can be considered an occupational dermatosis.^8^, ^10^

This case is peculiar due to the patients age group, the initial clinical presentation with micropustules, and the rarity of the etiological agent.

## Financial support

None declared.

## Authors' contributions

Hiram Larangeira de Almeida Jr.: Approval of the final version of the manuscript; design and planning of the study; drafting and editing of the manuscript; collection, analysis, and interpretation of data; effective participation in research orientation; intellectual participation in the propaedeutic and/or therapeutic conduct of the studied cases; critical review of the literature; critical review of the manuscript.

Luiz Roberto Kramer Costa: Approval of the final version of the manuscript; design and planning of the study; drafting and editing of the manuscript; collection, analysis, and interpretation of data; intellectual participation in the propaedeutic and/or therapeutic conduct of the studied cases; critical review of the literature; critical review of the manuscript.

Augusto Scott da Rocha: Approval of the final version of the manuscript; design and planning of the study; drafting and editing of the manuscript; collection, analysis, and interpretation of data; intellectual participation in the propaedeutic and/or therapeutic conduct of the studied cases; critical review of the literature; critical review of the manuscript.

## Conflicts of interest

None declared.
